# Small-window parametric imaging based on information entropy for ultrasound tissue characterization

**DOI:** 10.1038/srep41004

**Published:** 2017-01-20

**Authors:** Po-Hsiang Tsui, Chin-Kuo Chen, Wen-Hung Kuo, King-Jen Chang, Jui Fang, Hsiang-Yang Ma, Dean Chou

**Affiliations:** 1 Department of Medical Imaging and Radiological Sciences, College of Medicine, Chang Gung University, Taoyuan, Taiwan; 2 Medical Imaging Research Center, Institute for Radiological Research, Chang Gung University and Chang Gung Memorial Hospital at Linkou, Taoyuan, Taiwan; 3 Department of Medical Imaging and Intervention, Chang Gung Memorial Hospital at Linkou, Taoyuan, Taiwan; 4 Department of Otolaryngology–Head and Neck Surgery, Chang Gung Memorial Hospital and Chang Gung University, Taoyuan, Taiwan; 5 Department of Surgery, National Taiwan University Hospital, Taipei, Taiwan; 6 Taiwan Breast Cancer Foundation, Taipei, Taiwan; 7 Ph.D. Program in Biomedical Engineering, College of Engineering, Chang Gung University, Taoyuan, Taiwan; 8 Institute of Biomedical Engineering & Department of Engineering Science, University of Oxford, Oxford, UK

## Abstract

Constructing ultrasound statistical parametric images by using a sliding window is a widely adopted strategy for characterizing tissues. Deficiency in spatial resolution, the appearance of boundary artifacts, and the prerequisite data distribution limit the practicability of statistical parametric imaging. In this study, small-window entropy parametric imaging was proposed to overcome the above problems. Simulations and measurements of phantoms were executed to acquire backscattered radiofrequency (RF) signals, which were processed to explore the feasibility of small-window entropy imaging in detecting scatterer properties. To validate the ability of entropy imaging in tissue characterization, measurements of benign and malignant breast tumors were conducted (*n* = 63) to compare performances of conventional statistical parametric (based on Nakagami distribution) and entropy imaging by the receiver operating characteristic (ROC) curve analysis. The simulation and phantom results revealed that entropy images constructed using a small sliding window (side length = 1 pulse length) adequately describe changes in scatterer properties. The area under the ROC for using small-window entropy imaging to classify tumors was 0.89, which was higher than 0.79 obtained using statistical parametric imaging. In particular, boundary artifacts were largely suppressed in the proposed imaging technique. Entropy enables using a small window for implementing ultrasound parametric imaging.

Ultrasound backscattering and its relationship with the properties of scatterers in biological tissues is a widely pursued and critical topic in fundamental imaging research. In ultrasound imaging, the speckle results from the accumulation of random scatterings from within the resolution cell of the transducer^1^,^2^. On the basis of the randomness of ultrasound backscattered signals, backscattered envelope statistics (i.e., the echo amplitude distribution) can be modeled using mathematical statistical distributions to evaluate scatterer properties. Several statistical models have been proposed to describe the backscattered statistics for ultrasound tissue characterization, such as Rayleigh^2^, Rician^3^, K^4^, homodyned K^5^, generalized K^6^, Nakagami^7^, Nakagami compounding^8^,^9^,^10^, and McKay distributions^11^. General models that fit closely with different statistical distributions of backscattered envelopes are highly necessary.

Among the aforementioned models, the Nakagami distribution is the most frequently adopted model for tissue characterization because of its generality, simplicity, and low computational complexity^12^. Researchers reported potential applications of ultrasound Nakagami parametric imaging in visualizing backscattered statistics for quantifying the properties of tissues, such as in breast tumor classification^13^,^14^, liver fibrosis detection in rats^15^,^16^, radiotherapy evaluation^17^, cataract detection^18^, skin characterization^19^, vascular flow analysis^20^, thermal ablation monitoring^21^, and characterizing the structural anisotropy in the myocardium^22^. Various research groups demonstrated that the Nakagami image visualizes scatterer arrangements and concentrations and complements the conventional B-scan for tissue characterization.

Low spatial resolution is a substantial drawback of using statistical parameters, including the Nakagami parameter, to image tissues. The sliding window technique is a typical method for constructing ultrasound statistical parametric images^18^,^19^,^20^,^21^,^22^,^23^,^24^,^25^. A window within the image is used to collect local data for estimating a parameter; this estimation is repeated as the window is moved across the image, yielding a statistical parametric map. The window size determines the resolution of the parametric image: a smaller window offers a higher resolution. However, to avoid overestimating the statistical parameter, a window with a size corresponding to several times the spatial resolution of the B-scan is used to capture sufficient data points for calculation^26^,^27^. Hence, the resolution of statistical parametric images is lower than that of the conventional B-scan. In addition to low image resolution, ultrasound statistical parametric images entail another practical challenge: the data acquired for parameter estimation must follow the employed distribution model^28^,^29^. Not every imaging system outputs raw radiofrequency (RF) data of images. Adjusting the settings of an ultrasound system or using signal processing techniques may also alter the statistical distribution of the image data obtained from the system. In particular, different demodulation methods may cause different envelope statistics^30^, and different estimators yield different estimates^31^. These aspects highlight the lack of flexibility in applying statistical distributions to ultrasound parametric imaging.

Information entropy has a high potential for application in analyzing ultrasound backscattering. Shannon established a mathematical theory of communication and defined entropy as a measure of uncertainty in a random variable^32^. Hughes first proposed using information Shannon entropy for analyzing ultrasound signals and demonstrated that entropy can quantitatively describe microstructural changes in scattering media^33^,^34^,^35^,^36^. To visualize changes in the uncertainty of backscattered signals, ultrasound entropy imaging techniques were further developed^37^,^38^,^39^,^40^. Note that entropy is a function of probability density and thus is related to the distribution parameters^28^. However, the difference is that entropy is a relative measure of the signal uncertainty (a non-model-based statistical parameter) and therefore can be calculated using any type of data irrespective of the data distribution. In this case, using a large window to involve sufficient data points for a stable entropy calculation may be not a very critical consideration. We assume that using entropy affords the use of a small window for constructing high-resolution ultrasound parametric images. Using information entropy as a strategy for ultrasound small-window parametric imaging has not been explored previously.

In this study, we aim to (i) design an algorithmic scheme for ultrasound entropy imaging, (ii) investigate the feasibility of small-window entropy imaging in reflecting the scatterer properties, and (iii) validate the practical ability of small-window entropy imaging in tissue characterization by clinical measurements on breast tumors and comparisons with statistical parametric imaging based on the Nakagami distribution. The results showed that information entropy can be used to implement small-window parametric imaging (window side length (SL) =1 transducer pulse length) and to provide high-resolution images that visualize the uncertainty of backscattered RF signals for characterizing tissues. Compared with Nakagami parametric imaging, small-window entropy imaging improved the performance of breast tumor classification. This paper reports the potential contributions, impacts, and future work of entropy imaging in ultrasound tissue characterization.

## Results

Simulations and phantom experiments were conducted to explore the feasibility of small-window entropy imaging. Different types of phantoms were designed and the properties and materials of each type of phantom in the simulations and experiments are shown in Table 1. To confirm the practical performance of small-window entropy imaging in tissue characterization, clinical data of breast benign (fibroadenoma) and malignant (invasive carcinoma) tumors were further collected for validations.

### Simulations

**Figure 1:**
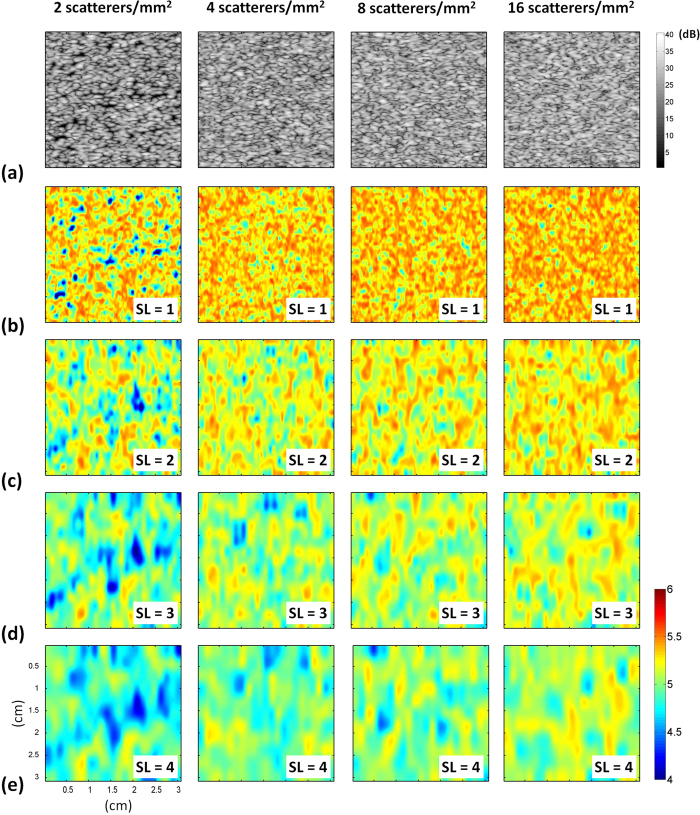
(**a**) B-mode and (**b–e**) entropy images of Type-I simulated phantoms with different number densities of scatterers. (**b**) SL = 1; (**c**) SL = 2; (**d**) SL = 3; (**e**) SL = 4. The dimensions for all images are the same. The grayscale and color bars represent the pixel values (the brightness) of the B-mode and entropy images, respectively. The shade of the entropy image depends on the window size.

**Figure 2:**
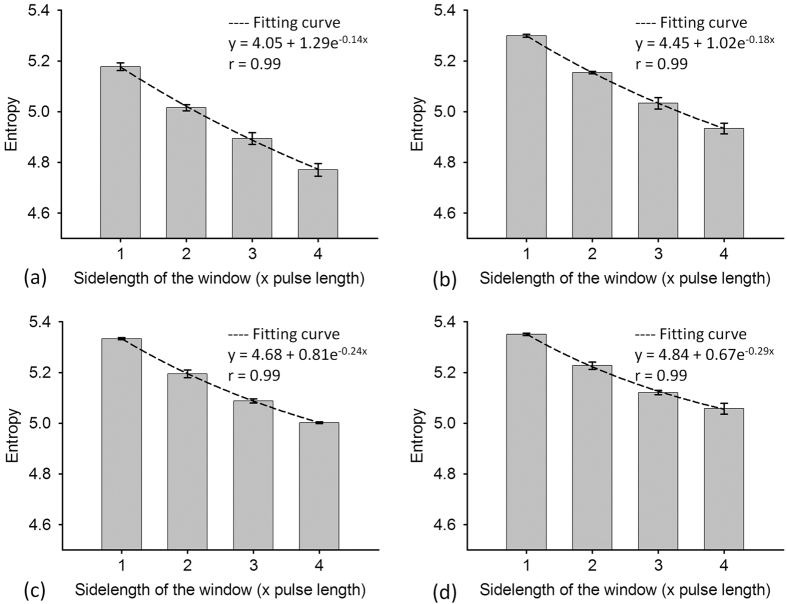
Simulated entropy values as a function of SL at different number densities of scatterers. (**a**) 2 scatterers/mm^2^; (**b**) 4 scatterers/mm^2^; (**c**) 8 scatterers/mm^2^; (**d**) 16 scatterers/mm^2^. At each scatterer concentration, the estimated entropy decreased with increasing SL. Data were expressed by mean ± standard deviation.

**Figure 3:**
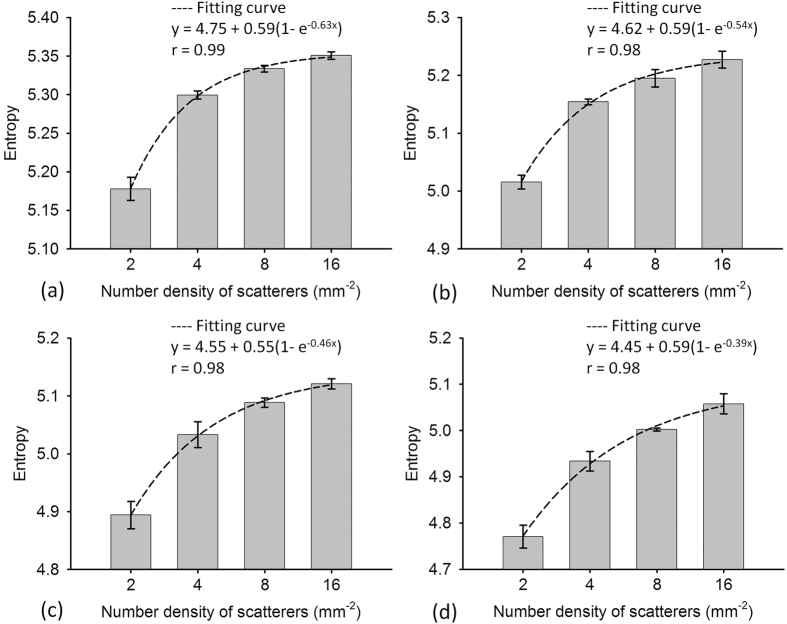
Simulated entropy as a function of the number density of the scatterers obtained at different SLs. (**a**) SL = 1; (**b**) SL = 2; (**c**) SL = 3; (**d**) SL = 4. Entropy images constructed using windows of different sizes can detect the variation in the number density of scatterers in a homogeneous medium. Data were expressed by mean ± standard deviation.

Figure 1 shows B-mode and entropy images of Type-I phantoms obtained using SLs of 1 to 4. The entropy image varied from blue–red-interlaced to red, which represents an increase in entropy, as the number density of scatterers increased from 2 to 16 scatterers/mm^2^. Figure 2 shows the entropy values as a function of SL at different number densities of scatterers. At each scatterer concentration, the estimated entropy decreased with increasing SL. Figure 3 shows entropy as a function of the number density of the scatterers obtained at different SLs. The entropy estimated from the entropy image constructed using SL = 1 increased from 5.17 ± 0.01 to 5.35 ± 0.005 as the number density of scatterers increased from 2 to 16 scatterers/mm^2^. This phenomenon was also observed for an SL of 2–4. Interestingly, entropy images constructed using a small window (SL = 1) can detect the variation in the number density of scatterers in a scattering medium.

**Figure 4:**
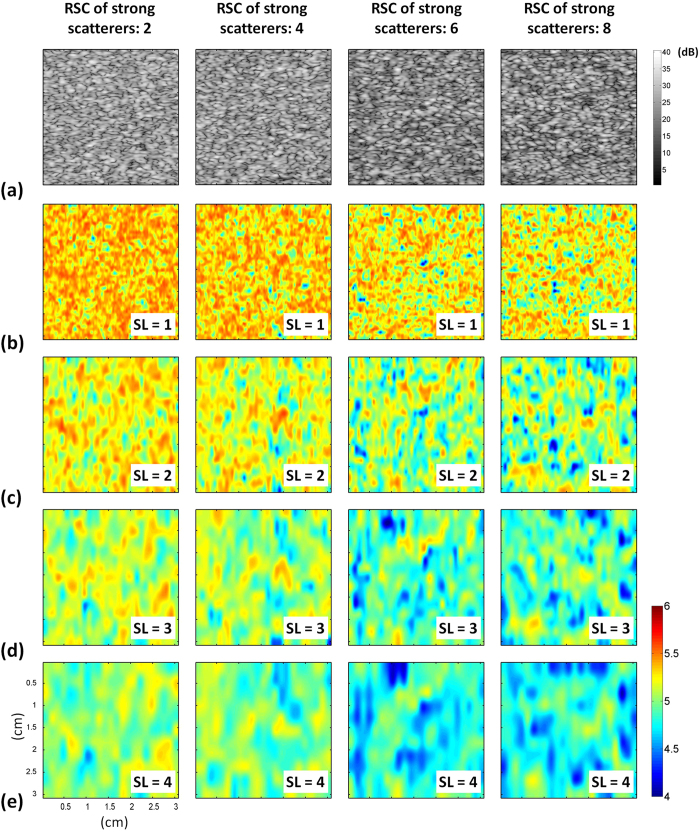
(**a**) B-mode and (**b–e**) entropy images of Type-II simulated phantoms containing strong scatterers with different RSCs constructed using different SLs. (**b**) SL = 1; (**c**) SL = 2; (**d**) SL = 3; (**e**) SL = 4. The dimensions for all images are the same. The grayscale and color bars represent the pixel values (the brightness) of the B-mode and entropy images, respectively. The change in the shade of the entropy images showed a decrease in the entropy because of the increasing phantom inhomogeneity.

**Figure 5:**
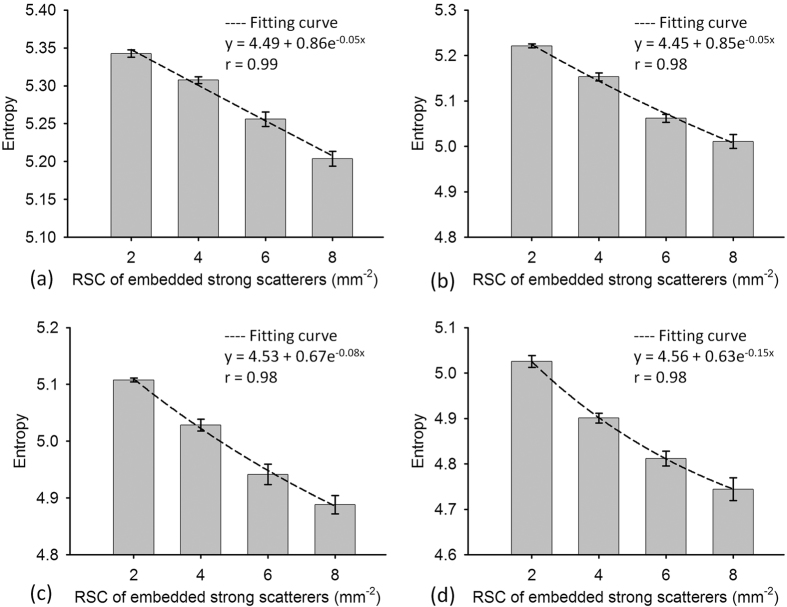
Simulated entropy as a function of the RSC of the embedded strong scatterers when different SLs were used. (**a**) SL = 1; (**b**) SL = 2; (**c**) SL = 3; (**d**) SL = 4. As the RSC of the strong scatterers increased, entropy in the image decreased. Data were expressed by mean ± standard deviation.

Figure 4 presents B-mode and entropy images of Type-II phantoms containing strong scatterers with different relative scattering coefficients (RSCs) constructed using different SLs. For SL = 1–4, the entropy image varied from red to red–blue-interlaced, which represents a decrease in entropy, as the RSC of the embedded strong scatterers increased from 2 to 8. Figure 5 presents entropy as a function of the RSC of the embedded strong scatterers when different SLs were used. As the RSC of the strong scatterers increased from 2 to 8, entropy in the image constructed using SL = 1 decreased from 5.34 ± 0.004 to 5.20 ± 0.009. Entropies obtained using SLs of 2–4 also decreased with increasing RSCs. The results showed that small-window entropy imaging (SL = 1) allows quantification of the tissue inhomogeneity.

**Figure 6:**
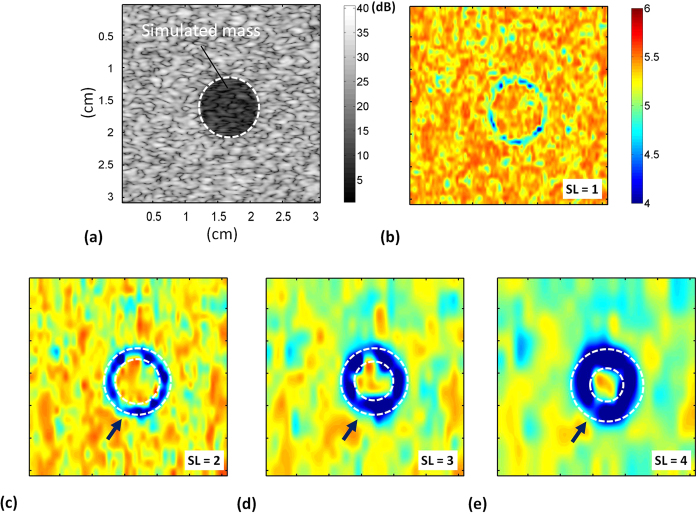
(**a**) B-mode and (**b–e**) entropy images of a simulated mass obtained using windows of different sizes. (**b**) SL = 1; (**c**) SL = 2; (**d**) SL = 3; (**e**) SL = 4. The dimensions for all images are the same. The grayscale and color bars represent the pixel values (the brightness) of the B-mode and entropy images, respectively. The boundary artifact was conspicuous when windows with a large SL were used, and it gradually diminished as the SL decreased from 4 to 1.

Figure 6(a–d) show B-mode and entropy images of a simulated mass (Type-III phantom) obtained using different window sizes. In the entropy image, the background and the mass had the same number density of scatterers but different RSCs. A blue image feature appeared at the background–mass interface (boundary artifact). The boundary artifact was conspicuous when windows with a large SL were used, and it gradually diminished as the SL decreased from 4 to 1. According to the above findings, the following results from the phantoms and clinical measurements were obtained using SL = 1 to investigate small-window entropy imaging in practical applications.

### Phantom experiments

**Figure 7:**
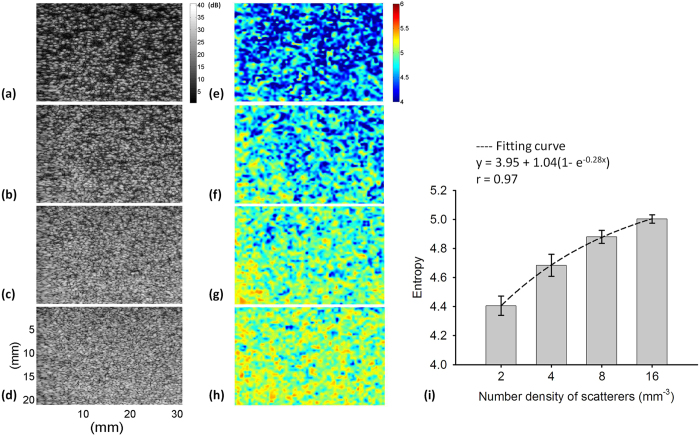
(**a–d**) B-mode images of Type-A agar phantoms with number densities of scatterers of 2, 4, 8, and 16 scatterers/mm^3^ (SL = 1; frequency = 6 MHz; scanned at a focus of 1 cm). The dimensions for all images are the same. The grayscale and color bars represent the pixel values (the brightness) of the B-mode and entropy images, respectively. (**e–h**) Entropy images corresponding to (**a–d**). (i) Entropy as a function of the number density of scatterers. Data were expressed by mean ± standard deviation.

**Figure 8:**
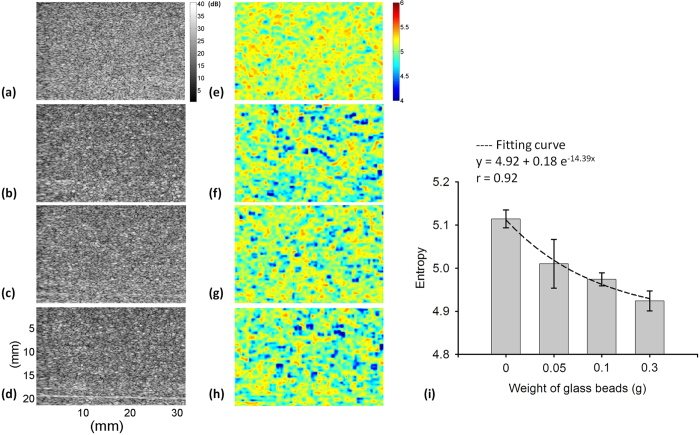
(**a–d**) B-mode images of Type-B agar phantoms to which 0, 0.05, 0.1, and 0.3 g of glass beads were added (SL = 1; frequency = 6 MHz; scanned at a focus of 1 cm). The dimensions for all images are the same. The grayscale and color bars represent the pixel values (the brightness) of the B-mode and entropy images, respectively. (**e–h**) Entropy images corresponding to (**a–d**). (i) Entropy as a function of the weight of glass beads. Data were expressed by mean ± standard deviation.

Figure 7 shows the B-mode and small-window entropy images of the Type-A phantom (frequency = 6 MHz; scanned at a focus of 1 cm). The entropy increased from 4.41 ± 0.07 to 5.00 ± 0.03 as the number density of scatterers increased from 2 to 16 scatterers/mm^3^. The results obtained from Type-A phantoms showed a similar trend with that of Type-I phantoms, indicating that entropy imaging can be used to visualize the variation in the number density of scatterers. Figure 8 displays the B-mode and small-window entropy images of the Type-B phantom (frequency = 6 MHz; scanned at a focus of 1 cm). The entropy decreased from 5.12 ± 0.02 to 4.91 ± 0.02 as the weights of the glass beads added in the background increased from 0 to 0.3 g. The experimental results for the Type-B phantoms confirmed the findings of the simulations of the Type-II phantoms: entropy allows the quantification of the tissue inhomogeneity.

**Figure 9:**
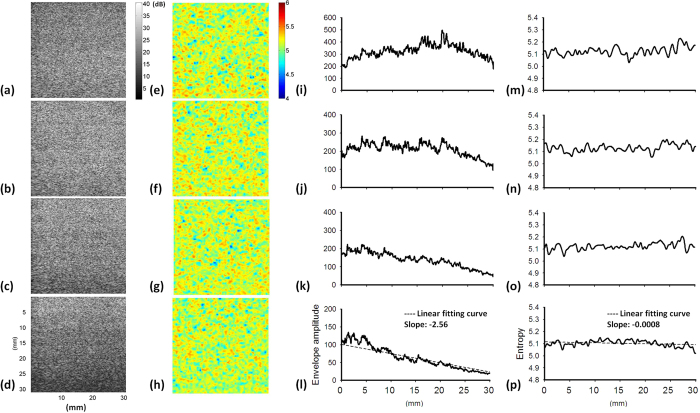
(**a–d**) B-mode images of Type-C agar phantoms to which lipid emulsions of 0%, 5%, 10%, and 20% were added (SL = 1; frequency = 6 MHz; scanned at a focus of 1 cm). The dimensions for all images are the same. The grayscale and color bars represent the pixel values (the brightness) of the B-mode and entropy images, respectively. (**e–h**) Entropy images corresponding to (**a–d**). (i) Entropy as a function of lipid concentration.

Figure 9(a–h) show the B-mode and small-window entropy images of the Type-C phantom (frequency = 6 MHz; scanned at a focus of 1 cm). Figure 9(i–l) illustrate the envelope amplitude values as functions of depth obtained by averaging all the envelope signals in Fig. 9(a–d), respectively. At a lipid concentration of 20%, the average attenuation rate for envelope signals was 2.56/mm, which was determined by the slope of the linear fitting curve. Figure 9(m–p) indicate that the average attenuation rate for the entropy image at a lipid concentration of 20% was 0.0008/mm, which is much lower than that of the B-scan.

**Figure 10:**
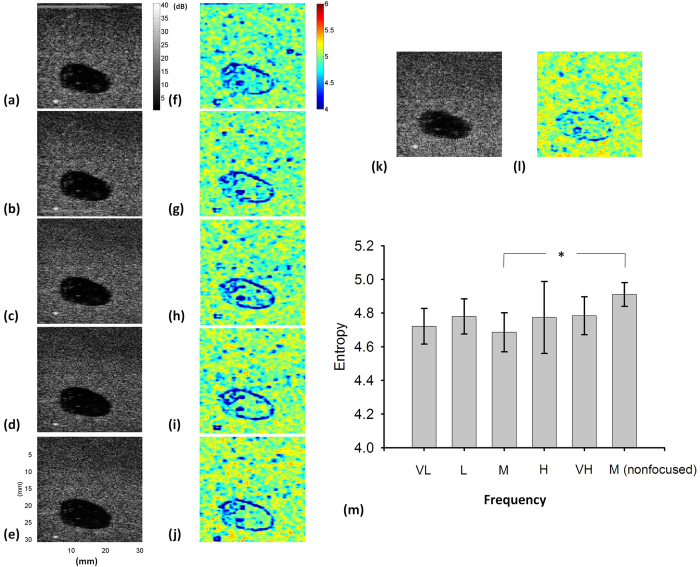
(**a–e**) B-mode images of the Type-D breast phantom with a mass at frequencies of 5, 5.5, 6, 7.5, and 8 MHz (SL = 1; scanned at a focus of 2 cm). (**f–j**) Entropy images corresponding to (**a–e**). (**k–l**) B-mode and entropy images scanned using 6 MHz and a focal length of 3 cm. The dimensions for all images are the same. The grayscale and color bars represent the pixel values (the brightness) of the B-mode and entropy images, respectively. (**m**) Entropy of the mass as a function of different settings (the “*” symbol indicates a *p* value < 0.05). Data were expressed by mean ± standard deviation.

Figure 10(a–j) display the B-mode and small-window entropy images of the Type-D phantom obtained using frequencies ranging from 5 to 8 MHz (scanned at a focus of 2 cm). Figure 10(k–l) show the results determined using a frequency of 6 MHz and a focal length of 3 cm (the mass was not located in the focal zone). Refer to Fig. 10(m). According to the *p* value obtained from the independent *t* test, increasing the frequency from 5 to 8 MHz did not result in a significant change in entropy, but the entropy estimated in the focal zone was lower than that obtained when the focal zone was moved away from the mass (*p* value < 0.05, denoted by the “*” symbol in the figure).

### Clinical data of breast tumor

**Figure 11:**
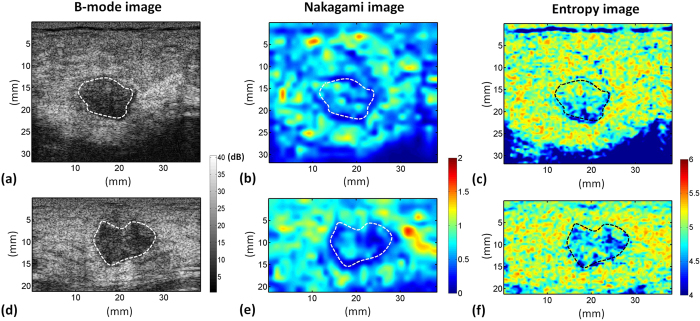
B-mode, Nakagami, and entropy (SL = 1) images of benign (**a–c**) and malignant (**d–f**) breast tumors. The shade of the Nakagami and entropy images of the malignant tumor was darker than that of the benign tumor.

**Figure 12:**
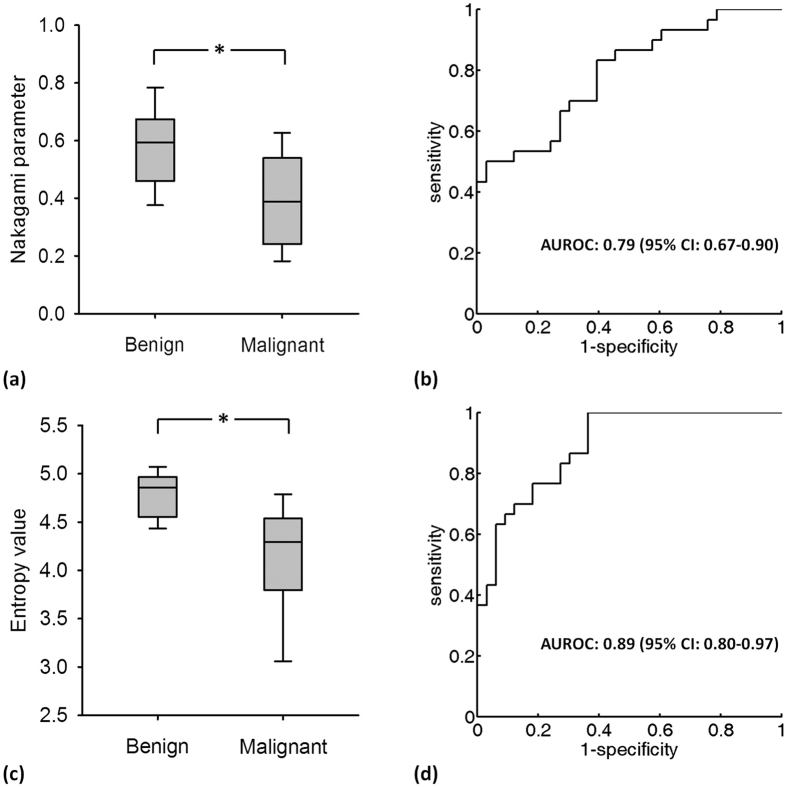
(**a**) The median Nakagami parameters for benign and malignant tumors were 0.59 (IQR: 0.46–0.67) and 0.46 (IQR: 0.28–0.55), respectively (*p* < 0.05). (**b**) ROC curve for using the Nakagami image to classify benign and malignant tumors. The AUROC was 0.75. (**c**) The median entropies for benign and malignant tumors were 4.86 (IQR: 4.57–4.96) and 4.37 (IQR: 4.01–4.61), respectively (the “*” symbol indicates a *p* value < 0.05). (**d**) ROC curve for using the entropy image to classify benign and malignant tumors. The AUROC was 0.82.

Figure 11 provides the B-mode, Nakagami, and small-window entropy images of benign and malignant breast tumors. The shade of the Nakagami and entropy images of the malignant tumor was darker than that of the benign tumor. Figure 12(a) shows that the median Nakagami parameter for the benign and malignant tumors was 0.59 (the interquartile range, IQR: 0.46–0.67) and 0.38 (IQR: 0.25–0.53), respectively (*p* < 0.05). Figure 12(b) displays the receiver operating characteristic (ROC) curve for using the Nakagami image to classify the benign and malignant tumors. The area under the ROC curve (AUROC) was 0.79 with a 95% confidence interval (CI) from 0.67 to 0.9. The accuracy was 69.84%, the sensitivity was 70%, and the specificity was 69.69%. Concurrently, the median entropy for the benign and malignant tumors, as shown in Fig. 12(c), was 4.86 (IQR: 4.57–4.96) and 4.29 (IQR: 3.87–4.51), respectively (*p* < 0.05). The AUROC for entropy imaging was 0.89 (95% CI: 0.80 to 0.97), and the accuracy was 79.36%, the sensitivity was 76.66%, and the specificity was 81.81%, as shown in Fig. 12(d). Table 2 compares performances of ultrasound small-window entropy and Nakagami imaging in classifying breast tumors. Compared with statistical parametric imaging constructed using a relatively large sliding window, small-window entropy imaging improved the performance of breast tumor classification.

## Discussion

### Significance of this study

This paper presents solutions to the problems associated with ultrasound statistical parametric imaging for visualizing the information associated with tissue microstructures. According to the simulation, phantom, and clinical measurement results obtained in this study, ultrasound entropy imaging is superior to statistical parametric mapping in the following aspects: (i) Small-window entropy images constructed using SL = 1 effectively describe the changes in scatterer properties. Compared with statistical parametric images, entropy images characterize tissues without sacrificing the resolution. (ii) Boundary artifacts occur at the interface when using sliding windows to construct parametric images. Because using entropy enables using a small window for parametric imaging, the effects of boundary artifacts are largely suppressed to improve the performance of tissue characterization. This is the first to demonstrate the usefulness of small-window entropy imaging in ultrasound tissue characterization.

### Effects of scatterer arrangements on entropy

Establishing a physical link between information entropy and the tissue microstructure is highly necessary for clinical applications. Clinically, normal soft tissue parenchyma, such those of the liver^41^ and breast^42^, may be treated as homogeneous media with a considerable number of randomly distributed scatterers. A change in the scatterer arrangement, from homogeneous to inhomogeneous, of a scattering medium may be used to explain the pathological change of a soft tissue from the normal to the abnormal stage. Our simulation and phantom results clarified the dependency of information entropy on scatterer properties (Figs 1, 2, 3, 4, 5, 7 and 8). In homogeneous media, entropy is determined by the number of scatterers. Increasing the number density of scatterers represents that more scatterers interact with the incident wave, thus complicating wave interference and generating backscattered signals with different amplitudes corresponding to high entropies (the signal uncertainty). In inhomogeneous media, a relatively high degree of variance in the scattering cross sections of the scatterers causes local variance in the amplitude of the RF signal waveform, thus narrowing the width of the signal probability distribution *w(y*); this condition reduces the estimated value of entropy.

### Effects of attenuation, frequency, and focus on entropy

It has been shown that entropy estimated using the probability distribution of ultrasound signals is proportional to ultrasound statistical parameters, and therefore it is expected to inherit the properties of statistical parameters, such as the dependencies of number density of scatterers, attenuation, noise, frequency, transducer focusing, and other factors that affect the size of the resolution cell^43^. The effects of number density of scatterers on entropy were discussed in the previous paragraph. The results showed that entropy value decreases with depth due to attenuation (Fig. 9), as supported by a recent report revealing that decreasing the signal amplitude with depth decrease statistical parameters^44^. The attenuation effect reduces the signal-to-noise ratio (SNR). Under a low SNR, noise typically behaves as a random variable with Gaussian distribution of zero mean, and the coupling of noise with backscattered echoes tends to change the signal amplitude distribution and the corresponding statistical parameters. Although the minimum requirement of a threshold SNR for entropy estimation is unknown currently, a previous study reported that estimations of statistical parameters can be stable and reach a steady state for SNRs above 20 dB, whereas the parameter estimation is SNR-dependent below 20 dB^45^. On the other hand, a significant dependence of entropy on frequency was not observed in the range of 5 to 8 MHz, but the transducer focus affects the entropy estimation (Fig. 10). This is largely due to that the degree of transducer focusing determines the size of the resolution cell, affecting the number of scatterers in the resolution cell that dominate the formation of backscattered signals and the corresponding estimations of statistical parameters^27^,^30^. Note that the transducer focusing simultaneously accompanies the diffraction effect in the far-field, which makes the resolution cell contain a large number of scatterers to result in overestimations of parameters for tissues with a low number density of scatterers^46^.

### Clinical exploration

The patterns of a breast parenchyma, which is composed of fatty and fibroglandular tissues, have been shown to be associated with the risk of developing breast cancer^47^. For healthy women, dense fibroglandular tissues are more common, and the corresponding density in a breast parenchyma decreases with age^48^. Ideally, a normal breast parenchyma based on fibroglandular tissues causes a fully developed speckle pattern in the B-mode image, which corresponds to the envelope statistics of the Rayleigh distribution^43^. Compared with normal breast tissue, benign fibroadenoma is composed of glandular tissues and local fibrous tissues or calcification^49^. Local fibrosis or calcification may cause local changes in the sound speed, density, and hardness, causing scatterers in a tumor to exhibit higher variability in the scattering cross sections^13^. Invasive carcinoma is the most prevalent malignant tumor. The cancer cells may spread to other parts of the body through the lymphatic system and bloodstream. In particular, malignant tumors may have diversified structures and calcification patterns^50^,^51^,^52^, such as (i) asymmetry and isolated dilated ducts^53^, (ii) calcifications with a greater hardness and density^51^, (iii) calcifications with irregular sizes, shapes, and nonuniform spatial distributions (e.g., branching or clustered)^54^, and (iv) stronger vascular flow and angiogenesis effects^55^,^56^. The aforementioned characteristics are expected to further strengthen the degree of variance in the echogenicity of scatterers, causing the entropic values of malignant tumors to be lower than those of benign tumors.

Refer to the results in Figs 11 and 12. Small-window entropy imaging is superior to statistical parametric imaging in classifying benign and malignant breast tumors. Prior to proposal of small-window entropy imaging, statistical parametric imaging has been widely used in breast tissue characterization^13^,^14^,^42^,^57^,^58^. However, some considerations and limitations exist when statistical parameters are used in practice. First, not every statistical distributions are applicable to characterizing breast tumors. For example, the estimation of the Nakagami parameter may be disturbed by the presence of structures in the breast^57^. This is why researchers attempted to use the mixture of distributions as a more general approach to describe the backscattered statistics^11^,^57^,^59^. Second, the estimation of the statistical parameter is affected by the used estimator. Moment-based estimators are frequently used for estimating statistical parameters. Nevertheless, using a maximum likelihood estimator can yield a smaller variance in estimations^60^. Although recent studies started to use different estimators for applications in tissue characterization^31^,^58^, the optimal estimator for parametric imaging of breast tumors has not been concluded. Third, as mentioned in Introduction, statistical parametric imaging needs the use of a large window to capture sufficient data points for stable parameter estimations. A significant boundary artifact will occur in the parametric image to degrade the performance in classifying breast tumors^13^.

Compared with statistical parameters, entropy enables using a small window for implementing ultrasound parametric imaging. In this condition, boundary artifacts can be largely suppressed to improve the classification of breast tumors (Fig. 12), as discussed below.

### Suppression of boundary artifacts

Boundary artifacts frequently appear at the interfaces of tissues in ultrasound parametric imaging^13^ because the existence of edges and boundaries affects the backscattered statistics^57^,^59^. When a sliding window moves across the interface during parametric imaging, the window covers not only the backscattered data from the interface but also those from the background tissues. The difference in the echo amplitude of the interface and background tends to narrow the probability distribution of the data, causing the parameter to be underestimated and generating a boundary artifact. Among all possibilities, using a small window for parametric imaging is the simplest approach to suppress the boundary artifacts. Information entropy is a relative measure of the signal uncertainty, not a model-based parameter or an absolute physical estimate. Therefore, unlike the distribution parameters, entropy allows calculation using less data points acquired from a small window as long as its detectability in the properties of scatterers can be obtained. This study demonstrated that entropy combined with the small-window technique can implement parametric imaging to characterize tissues without significant boundary artifacts (Figs 6 and 11).

## Conclusions

Computer simulations and phantom experiments were conducted to investigate ultrasound small-window entropy imaging and its performance in detecting changes in the properties of scatterers. Small-window entropy imaging constructed using SL = 1 effectively visualizes changes in the number density of scatterers and inhomogeneity without significant boundary artifacts. Clinical measurements on breast tumors also showed the usefulness of small-window entropy imaging in practical tissue characterization. Information entropy enables using a small window for implementing high-resolution ultrasound parametric imaging.

## Materials and Methods

### Simulations

Two-dimensional (2D) computer simulations were executed at a sampling rate of 50 MHz and a sound speed of 1540 m/s to generate image RF data; previous studies have detailed the simulation method^31^,^43^,^61^. A 5-MHz Gaussian pulse (a pulse length of 0.89 mm, a bandwidth of 80%, and a beam width of 1.66 mm) was generated as the incident wave with a 2D resolution cell with an area of approximately 1.48 mm^2^ (0.89 mm × 1.66 mm). The computer phantom *Z* is a 2D matrix with randomly positioned delta functions that describe the spatial arrangement of *K* scatterers; it is given by


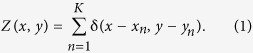


Simulated image RF data were obtained by convoluting the incident wave with the computer phantoms.

A total of five phantoms with sizes 3 cm × 3 cm were constructed for each number density of scatterers (2, 4, 8, and 16 scatterers/mm^2^). A computer phantom with 16 scatterers/mm^2^ has approximately 24 scatterers per resolution cell (>10 scatterers per resolution cell), which is sufficient to produce the fully-developed speckle B-mode image (the backscattered statistics follow Rayleigh distribution)^30^. In this study, we term these phantoms Type-I phantoms to simulate changes in the backscattered statistics from pre-Rayleigh to Rayleigh distributions. The magnitude of the delta function is 1 and is considered the relative RSC for each scatterer in the phantom.

To simulate an inhomogeneous medium with different degrees of scatterer echogenicity variance, we added randomly distributed strong scatterers at a number density of 1 scatterer/mm^2^ in each Type-I phantom; these Type-I phantoms containing strong scatterers are defined as Type-II phantoms. The RSCs of the strong scatterers were adjusted by multiplying weight factors and the delta functions. Thus, Type-II phantoms *Z*_s_ are expressed as


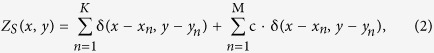


where *c* (=2, 4, 6, and 8) is the weight factor used to simulate the RSCs of strong scatterers, and *M* represents the number of strong scatterers. For each weight factor, five Type-II phantoms were produced for signal formation and analysis. Adjusting the RSC was not used for simulating the properties of real tissues. This is a method used to generate ultrasound signals with changes in the backscattered statistics from Rayleigh to pre-Rayleigh distributions, as proposed in our previous study^31^. Based on the suggestion given in a previous study^30^, strong scatterers contribute amplitude values 4 times the mean amplitude of random scatterers. Therefore, the range of the weight factor *c* from 2 to 8 was chosen to simulate variations in the RSCs of strong scatterers.

The existence of edges and boundaries in B-scan images alters the statistics of the backscattered echo^59^. Therefore, we created Type-III phantoms, each containing a background material and an embedded cylindrical object with a diameter of 1 cm. Both the background and the cylindrical object have the same number density of scatterers (16 scatterers/mm^2^). The RSCs of the scatterers in the background and the cylindrical object were set to 1 and 0.1, respectively. The Type-III phantoms were generated to simulate the mass to explore the boundary artifact of the entropy image corresponding to the interface between the tissue background and the target.

Note that the effects of frequency, diffraction due to transducer focusing, and attenuation were not considered in the simulation model. To explore performances of ultrasound entropy imaging in scatterer characterization under frequency, diffraction, and attenuation effects, phantom experiments were further conducted.

### Phantom experiments

The phantoms were constructed by boiling and cooling agar–water mixtures (dissolving 3 g of the agar powder into 200 mL of water) and adding different materials, including glass beads with diameters of 75 μm (Model 59200U, Supelco, Bellefonte, PA, USA), graphite powder with diameters < 20 μm (Model 282863, Sigma-Aldrich, St. Louis, MO, USA), and soybean-oil lipid emulsions (Intrafat, Nihon Pharmaceutical Industry, Osaka, Japan). Both the glass beads and graphite powder were used as acoustic scatterers in the scattering medium, and the materials of the lipid emulsions were used to produce an acoustic attenuation effect in the phantoms^62^. Three types of phantoms were constructed, namely Type-A, Type-B, and Type-C. A tissue-mimicking breast phantom (Model BPB170, Blue Phantom, Redmond, WA, USA) was used as a Type-D phantom to provide a simulated mass.

The properties and materials of each type of phantom are shown in Table 1. For Type-A phantoms, the number densities of scatterers (NDS) were determined by


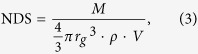


where *M, r*_*g*_, and *ρ* correspond to the mass, radius, and density of the glass beads, respectively, and *V* denotes the volume of the agar phantom. The number densities of scatterers for Type-A phantoms were set the same as those in the simulations (2 to 16 scatterers/mm^3^). For Type-B and C phantoms, a large amount of graphite powder is required to be added for the formation of significant backscattered signals because graphite powders with small diameters (<20 μm) are relatively weak scatterers. Thus, the same number densities of scatterers are not applicable in Type-B and C phantoms. Alternatively, the scatterer concentration (the ratio of powder weight to the volume of the phantom) was used. Using equation (3) and assuming that the diameter of graphite powder is 20 μm, the number density of scatterers in the background of the phantom (made using 2 g graphite powder and 200 mL water) is estimated to be at least 1000 scatterers/mm^3^. This estimated number density ensures that a large number of randomly distributed scatterers exist in the resolution cell, making backscattered envelopes obey the Rayleigh distribution.

Five independent scans of each phantom were performed using a commercial ultrasound imaging system (Model 3000, Terason, Burlington, MA, USA) with a linear array transducer (Model 10L5, Terason). The Terason scanner enables RF data access and frequency selection. The transmitting central frequency can be set at approximately 5, 5.5, 6, 7.5, and 8 MHz (denoted by VL, L, M, H, and VH, respectively) by using the software, and the pulse-echo tests of the transducer in the laboratory showed that the pulse length of the transducer was approximately three times the wavelength. In the experiments on the Type-A, -B, and -C phantoms, the image RF data consisted of 128 A-lines of backscattered signals, which were acquired using the same transmitting frequency (6 MHz) and a focus of 1 cm to investigate the dependency of entropy on the number density of scatterers, the degree of variance in the echogenicity, and the attenuation effect. In the experiments on the Type-D phantom, different transmitting frequencies and focus settings were used to explore the effects of frequency and focus on ultrasound entropy imaging.

### Clinical measurements

Clinical data were collected for preliminarily validating ultrasound entropy imaging for characterizing tissues. This study was approved by the Institutional Review Board of National Taiwan University Hospital, and signed informed consent forms were obtained from the patients. All the experimental methods were carried out in accordance with the approved guidelines. At the Department of Surgery, female patients that required clinical biopsy examinations of masses were recruited. Breast tumors were classified as benign or malignant by a pathologist according to the biopsy reports. Prior to the biopsy examination, ultrasound scanning of the patients was performed by the same sonographer to reduce the inter-rater variability of ultrasound scan. For each patient, five scans were performed to collect grayscale B-mode images and the corresponding RF backscattered data using the same Terason system and linear array transducer for manual tumor contour segmentation and entropy imaging, respectively. Images of tumors with incomplete contours caused by posterior shadow effect were excluded in the analysis. Under the above criterion, a total of 63 patients (*n* = 63) were recruited, including 33 benign (fibroadenoma) and 30 malignant (invasive carcinoma) masses.

### Entropy estimation

Shannon proposed the information entropy to quantify information^32^,^63^. In ultrasound imaging, the Shannon entropy of backscattered RF signals *f(t*) is defined as the negative of the logarithm of the backscattered probability distribution *w(y*)^34^:


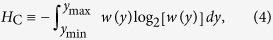


where *y*_min_ and *y*_max_ represent the minimal and maximal values of *f(t*), respectively. In this study, the statistical histogram of RF signals was used to represent *w(y*)^28^,^40^. Practically, Shannon entropy is obtained as a discrete form of equation (4) using the digitized versions of the underlying continuous waveform. Entropy is a measure of the uncertainty or unpredictability of ultrasound backscattered signals.

### Algorithmic scheme of entropy imaging

**Figure 13:**
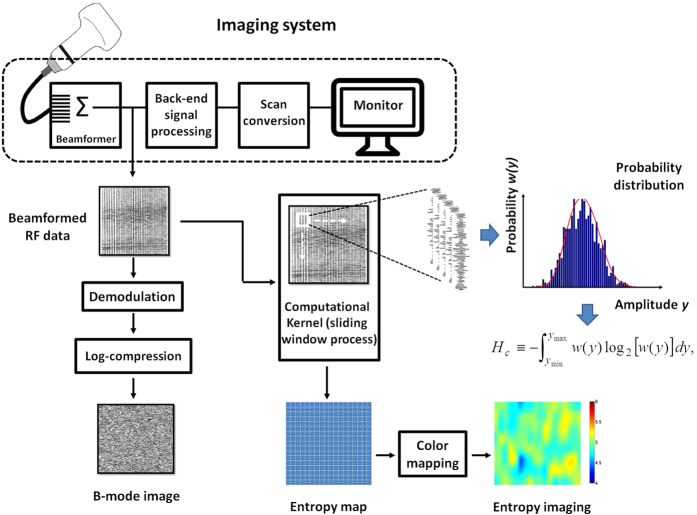
The algorithmic scheme designed for constructing the B-mode and information entropy images using the backscattered signals. The B-mode image was formed using a logarithm-compressed envelope image. The image RF data were used for entropy estimation and imaging using a standard sliding window algorithm.

Figure 13 shows the algorithmic scheme designed for constructing the B-mode and information entropy images using the backscattered signals. The algorithm was programmed using MATLAB software (version R2012a, The MathWorks, Inc., MA, USA). The envelope image was constructed using the absolute value of the Hilbert transform to demodulate each scan line, and the B-mode image was formed using a logarithm-compressed envelope image at a dynamic range of 40 dB.

Concurrently, the beamformed RF data were used for entropy estimation and imaging using a standard sliding window algorithm, and the entropic parametric map was constructed through the following two steps: (i) a square window within the image data was used to acquire local RF signals for establishing the probability density function *w(y*) and calculating the entropy value using equation (4), which was assigned as the new pixel located in the center of the window; and (ii) the window was moved across the entire range of image data in steps of the number of pixels corresponding to a window overlap ratio, and step 1 was repeated to yield the entropy parametric map. A low window overlap ratio results in a low line density of a parametric image. The decreased line density decreases the spatial resolution of an image^64^, making spatial information insufficient to describe the region of interest (ROI). A high window overlap ratio results in a high line density. However, the computational efficiency and speed may reduce because a large amount of data must be processed. A recent study showed that the window overlap ratio does not affect ultrasound parametric imaging and estimations of statistical parameters^61^. To have a trade-off between the image resolution and the computational time, a 50% window overlap ratio was used.

### Data analyses for simulation

To explore the effect of the window size on the entropy image, the entropic parametric map corresponding to each image data was constructed using square sliding windows with SLs ranging from 1 to 4 times the transducer pulse length (denoted as SL = 1, 2, 3, and 4). For each simulated entropy image, the pixel data were acquired from an ROI with a size of 5 × 5 mm^2^ to explore the entropy value as a function of the SL, number density of the scatterers, and RSC of the embedded strong scatterers. The trends of data were described by curve fitting, and data were expressed by mean ± standard deviation.

### Data analyses for phantom experiments

SL = 1 was used for entropy imaging of phantom data according to the findings in the simulations (please see the section of Results). ROIs (5 × 5 mm^2^) located in the focal zone were applied to the phantom results to investigate the effects of scatterer properties, attenuation, frequency, and focus on entropy values. Data were expressed by mean ± standard deviation. To evaluate statistical significance, an independent *t* test was performed to calculate the probability value *p*.

### Data analyses for clinical data

The entropy images (SL = 1) of breast tumors were analyzed according to the ROIs that were manually determined by the surgeon, who was blind to the biopsy reports. To compare the performance of entropy imaging with that of statistical parametric imaging in clinical breast tumor characterization, Nakagami images of each patient were also constructed and the same ROIs determined by the surgeon were applied to the Nakagami images for analysis. The algorithm of Nakagami imaging was based on the sliding window processing, which was the same technique as that described in Fig. 13. The envelope image was obtained from the absolute value of the Hilbert Transform of the RF data. A Nakagami image was constructed using a square window (SL = 3; a 50% window overlap ratio) sliding on the envelope image for collecting local envelope data *R* and estimating local Nakagami parameters *m* by


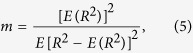


SL = 3 was used because the side length of the window corresponding to three times the pulse length of the transducer enables a stable estimation of the Nakagami parameter^13^,^15^,^25^,^29^. The Nakagami parameters and the entropy values of benign and malignant tumors were shown in the box plots (expressed by the median and interquartile range, IQR) for comparisons. For evaluating statistical significance, an independent *t* test was performed to calculate the *p* value. The ROC curve was used to evaluate the clinical performances of the Nakagami and entropy images in classifying benign and malignant tumors. Statistical analysis was performed sing SigmaPlot (version 9.0, Systat Software, Inc., CA, USA).

## Additional Information

**How to cite this article:** Tsui, P.-H. *et al*. Small-window parametric imaging based on information entropy for ultrasound tissue characterization. *Sci. Rep.* **7**, 41004; doi: 10.1038/srep41004 (2017).

**Publisher's note:** Springer Nature remains neutral with regard to jurisdictional claims in published maps and institutional affiliations.

## Figures and Tables

**Table 1:** Properties and materials of each type of phantom in the simulations and experiments.

	Type no.	Objectives	Properties of the scatterers in the background	Additional scatterers or additive in the background
Simulations	Type-I	Simulating changes in the number density of scatterers	Point scatterers (RSC: 1) Number density of scatterers: 2, 4, 8, and 16 scatterers/mm^2^	—
	Type-II	Simulating changes in the degree of variance in the echogenicity	Point scatterers (RSC: 1) Number density of scatterers: 16 scatterers/mm^2^	Point scatterers (RSC: 2, 4, 6, and 8) Number density of scatterers: 1 scatterers/mm^2^
	Type-III	Simulating the tissue interface	Point scatterers (RSC: 1) Number density of scatterers: 16 scatterers/mm^2^	An embedded cylindrical object with point scatterers (RSC: 0.1) Number density of scatterers: 16 scatterers/mm^2^
Phantom experiments	Type-A	Simulating changes in the number density of scatterers	Glass beads Number density of scatterers: 2, 4, 8, and 16 scatterers/mm^3^	—
	Type-B	Simulating changes in the degree of variance in the echogenicity	Graphite powder Scatterer concentration: 2 g in 200 mL water (>1000 scatterers/mm^3^)	Glass beads Scatterer concentration: 0.05, 0.1, and 0.3 g in 200 mL water
	Type-C	Simulating the attenuation effect	Graphite powder Scatterer concentration: 2 g in 200 mL water (>1000 scatterers/mm^3^)	Soybean-oil lipid emulsions Lipid concentration: 0%, 5%, 10%, and 20%
	Type-D	Simulating the tissue interface and exploring effects of frequency and focus	A tissue-mimicking breast phantom	—

RSC: Relative scattering coefficients of scatterers in the simulations. The phantoms were constructed by boiling and cooling agar–water mixtures (dissolving 3 g of the agar powder into 200 mL of water) and adding different materials, including glass beads with diameters of 75 μm (Model 59200U, Supelco, Bellefonte, PA, USA), graphite powder with diameters < 20 μm (Model 282863, Sigma-Aldrich, St. Louis, MO, USA), and soybean-oil lipid emulsions (Intrafat, Nihon Pharmaceutical Industry, Osaka, Japan). In the simulations and phantom experiments, scatterers were randomly distributed.

**Table 2:** Performance comparisons between ultrasound small-window entropy and Nakagami imaging in classifying benign and malignant breast tumors.

Methodology		Small-window ultrasound entropy imaging	Nakagami statistical parametric imaging
Median (IQR) of the Nakagami parameter	Benign	4.86 (4.57–4.96)	0.59 (0.46–0.67)
	Malignant	4.29 (3.87–4.51)	0.38 (0.25–0.53)
Dynamic range of the parameter		1.73–5.09	0.24–0.87
Cutoff value		4.52	0.47
Sensitivity%		76.66%	70.00%
Specificity%		81.81%	69.69%
Accuracy%		79.36%	69.84%
LR+		4.21	2.31
LR−		0.28	0.43
PPV%		79.31	67.74
NPV%		79.41	71.87
AUROC (95% CI)		0.89 (0.80–0.97)	0.79 (0.67–0.90)

LR+: positive likelihood ratio, LR−: negative likelihood ratio, PPV: positive predictive value, NPV: negative predictive value, AUROC: area under the receiver operating characteristics curve.
